# Additive manufactured, highly resilient, elastic, and biodegradable poly(ester)urethane scaffolds with chondroinductive properties for cartilage tissue engineering

**DOI:** 10.1016/j.mtbio.2020.100051

**Published:** 2020-04-13

**Authors:** S. Camarero-Espinosa, C. Tomasina, A. Calore, L. Moroni

**Affiliations:** a MERLN Institute for Technology-inspired Regenerative Medicine, Complex Tissue Regeneration Department, Maastricht University, P.O. Box 616, 6200MD, Maastricht, the Netherlands; b Polyganics, Rozenburglaan 15A, 9727 DL, Groningen, the Netherlands; c Aachen Maastricht Institute for Biobased Materials, Maastricht University, P.O. Box 616, 6200 MD, Maastricht, the Netherlands

**Keywords:** Fused deposition modelling, Chondrogenesis, Stem cell, Bioresorbable, Tissue regeneration

## Abstract

Articular cartilage was thought to be one of the first tissues to be successfully engineered. Despite the avascular and non-innervated nature of the tissue, the cells within articular cartilage – chondrocytes – account for a complex phenotype that is difficult to be maintained in vitro. The use of bone marrow–derived stromal cells (BMSCs) has emerged as a potential solution to this issue. Differentiation of BMSCs toward stable and non-hypertrophic chondrogenic phenotypes has also proved to be challenging. Moreover, hyaline cartilage presents a set of mechanical properties – relatively high Young's modulus, elasticity, and resilience – that are difficult to reproduce. Here, we report on the use of additive manufactured biodegradable poly(ester)urethane (PEU) scaffolds of two different structures (500 μm pore size and 90° or 60° deposition angle) that can support the loads applied onto the knee while being highly resilient, with a permanent deformation lower than 1% after 10 compression-relaxation cycles. Moreover, these scaffolds appear to promote BMSC differentiation, as shown by the deposition of glycosaminoglycans and collagens (in particular collagen II). At gene level, BMSCs showed an upregulation of chondrogenic markers, such as collagen II and the Sox trio, to higher or similar levels than that of traditional pellet cultures, with a collagen II/collagen I relative expression of 2–3, depending on the structure of the scaffold. Moreover, scaffolds with different pore architectures influenced the differentiation process and the final BMSC phenotype. These data suggest that additive manufactured PEU scaffolds could be good candidates for cartilage tissue regeneration in combination with microfracture interventions.

## Graphical abstract

**Figure:**
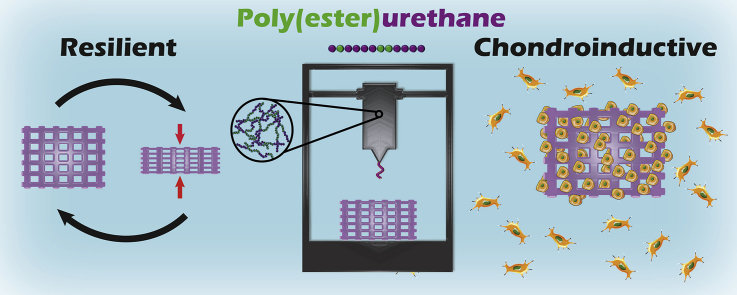

## 1 Introduction

Hyaline cartilage is the connective tissue present at the end of long bones, serving as a cushion and allowing for frictionless movement upon articulation [1,2]. The cartilage has poor self-healing properties. Damage to the tissue, by trauma or as a consequence of degenerative diseases, results in the evolution of the formed defect until the subchondral bone is reached, leading in many cases to disability of the patient. Clinical treatments to regenerate cartilage are based on the expansion of autologous chondrocytes and later implantation on the defect area, with or without the aid of a matrix support (autologous chondrocyte implantation [ACI] or matrix-assisted ACI [MACI]), or the recruitment of bone marrow–derived stem cells (BMSCs) from the subchondral bone [3]. In both cases, cells invading the defect form a de-novo tissue that differs from the native one morphologically, histochemically, and, most importantly, biomechanically. While these methods result in an initial pain relief for the patient, at long term these fail to regenerate a functional tissue [3,4].

Avascular and non-innervated in nature, articular cartilage was expected to be one of the first tissues to be successfully engineered in vitro [5,6]. However, autologous chondrocytes have low availability (1–5% of total tissue volume) and low proliferative character, and in vitro expansion leads to the dedifferentiation of the cells [7]. Thus, the use of BMSCs has emerged as an optimal solution to these limitations. Differentiation of BMSCs toward chondrogenic phenotypes in hydrogels and pellet cultures has been largely studied [[8], [9], [10]]. However, while these systems recapitulate the events observed during in vivo endochondral bone formation, they lack sufficient support to withstand the high and repetitive loads applied onto the knee. Thus, research has been focused on developing chondroinductive materials and biofabrication processes that allow generation of scaffolds which are able to promote chondrogenic differentiation of BMSCs and withstand the cyclic loads applied on the tissue [11].

Hydrogel systems have been extensively studied because of their intrinsic capability to maintain a rounded cellular structure that favors chondrogenic phenotypes and their highly hydrated state similar to that of the cartilage (80 wt% water) [8]. These have been fabricated out of naturally derived materials such as alginate, silk, and collagen, as well as synthetic polymers such as poly(ethylene glycol) [12]. Hydrogels usually present a high resilience, similarly to the native tissue, but account for a reduced Young's modulus. Therefore, many efforts have been focused on the reinforcement of such materials with fillers or fibrous structures [[13], [14], [15], [16], [17], [18]]. Others have explored various scaffold fabrication techniques such as salt leaching, gas foaming, or thermally induced phase separations to create porous scaffolds that can host the cells while providing a higher mechanical integrity [[19], [20], [21]]. The use of fibrous scaffolds fabricated via spinning has also been investigated with relative success due to the difficulty to generate 3D scaffolds and the low-porosity nature that hinders cell infiltration [22,23].

Fused deposition modeling (FDM) has emerged on the last decades as a rapid and patient-centered biofabrication technique that allows for the layer-by-layer deposition of a great variety of materials. The use of this additive manufactured scaffold for cartilage tissue engineering or regeneration has shown great promise because of the flexibility of the technique on materials, structures, and mechanical properties of the generated scaffolds [24]. Traditional polyesters used in FDM such as polycaprolactone and poly(lactic acid) or polyether-based elastomers, such as poly(ethylene oxide) terephthalate/poly(butylene) terephthalate, have shown success on in vitro engineering of the articular cartilage [[25], [26], [27], [28]]. However, these materials generally present a high Young's modulus but a rather low elasticity, yield strain and resilience [29,30]. To tackle this problem, researchers have combined hydrogels with polyester-based FDM scaffolds. These composite scaffolds present the capability of supporting cell chondrogenesis, while supporting the loads applied onto the knee [16,31]. The use of materials that offer high compressive strength while being highly resilient, such as poly(ester)urethanes , has so far been limited to solvent-based biofabrication techniques [[32], [33], [34], [35], [36], [37]]. Zuidema et al. [38] reported on the in vitro biodegradation of solvent-based fabricated PEU foams based on a prepolymer of 50 mol% dl-lactide (50/50 D/L ratio) and 50 mol% ε-caprolactone mixture that was later chain extended with a urethane hard segment (BDI-BDO-BDI-BDO-BDI). The degradation products of this PEU have proven biocompatible in vitro, and a PEU based on the same building blocks of hard segment but with a shorter length (BDO-BDI-BDO), has proven bioresorbable in a 3-year subcutaneous implantation study in rats and rabbits [39,40]. We have recently reported the use of FDM of biodegradable PEUs of the same composition as from the study by Zuidema et al. [38] for cartilage tissue engineering [41]. Additive manufacturing offers the possibility of creating scaffolds with different internal structure or mesh. Variations on the pore size and shape of the mesh have shown to exert an effect on the mechanical properties of the final object and on the differentiation potential of the cells cultured within these [26,[41], [42], [43]]. The fabricated PEU scaffolds showed an extended elastic region with yield strains of up to 50% and a Young's modulus that can be tuned with the scaffold architecture. These materials supported the redifferentiation of dedifferentiated chondrocytes even in basal conditions (no growth factors or chondroinductive molecules added to the media) and performed better than traditional pellet cultures. Here, we report on the additive manufacturing of highly resilient PEU scaffolds with printing patterns of 90° and 60°, of the same chemical characteristics, that support the differentiation of BMSCs toward a chondrogenic phenotype.

## 2 Materials and methods

### 2.1 Materials

The biodegradable PEU used for this study was synthesized and kindly provided by Polyganics B.V. in the form of pellets and was used as received. The PEU was synthesized from a random, polyester prepolymer soft segment that was chain extended with a hard urethane segment [44]. In brief, the soft segment prepolymer was prepared by ring opening polymerization of a 50 mol% dl-lactide (50/50 D/L ratio) and 50 mol% ε-caprolactone mixture with butanediol as the initiator. The prepolymer (Mn = 2000 g/mol) was then chain extended with a uniform 5-block urethane hard segment (BDI-BDO-BDI-BDO-BDI), where BDI and BDO stand for 1,4- butanediisocyanate and 1,4-butanediol, respectively.

### 2.2 Fused deposition modeling

**Fig. 1:**
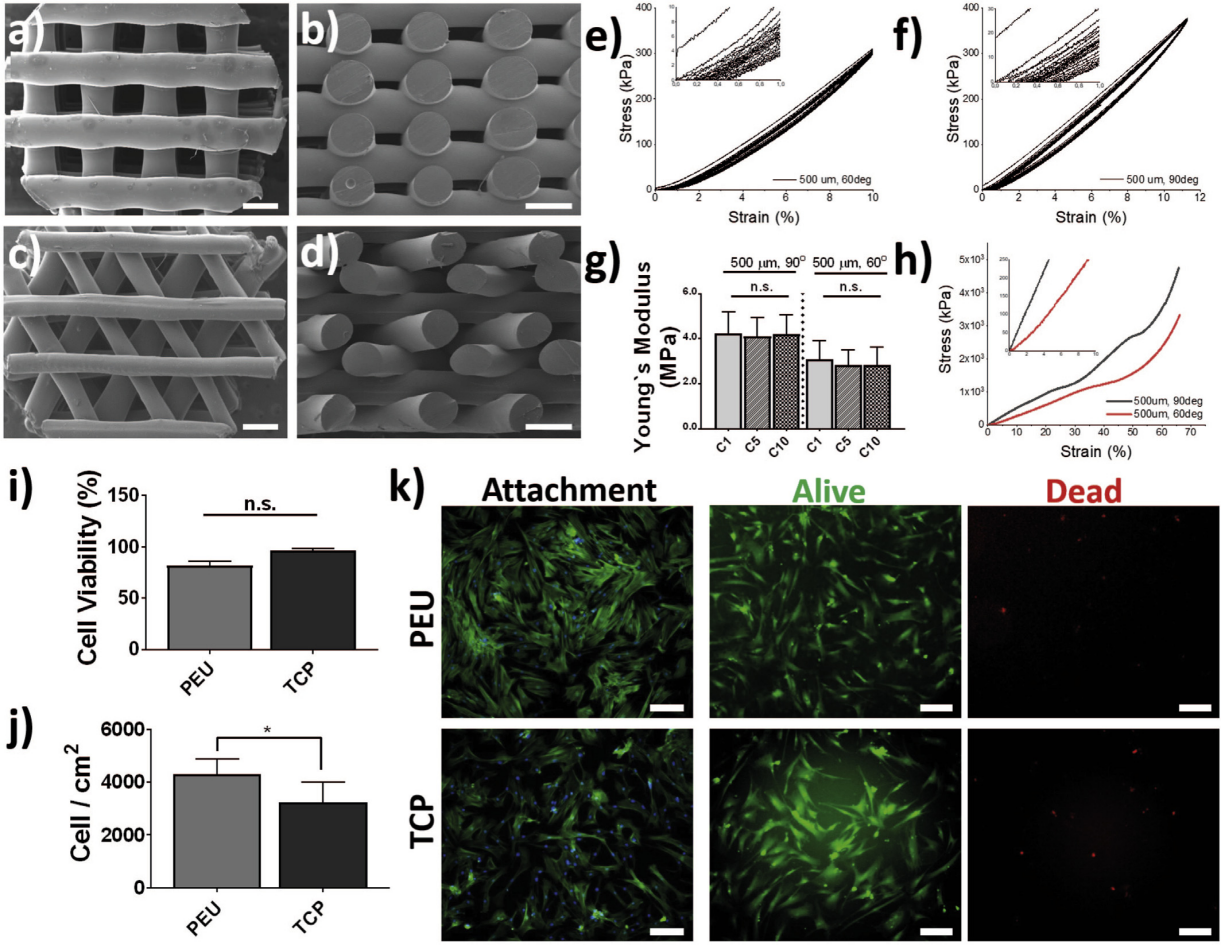
Structural, mechanical, and biocompatibility characterization. Scanning electron microscopy images of (a and b) scaffolds with 500 μm and 90° and (c and d) 500 μm and 60° deposition pattern under top (a and c) and side (b and d) views. Scale bars are 500 μm. Cyclic compression of scaffolds fabricated with 60° (e) and 90° (f) pore angles. Insets show zoom-in of the 0–1% strain range. (g) Young's modulus of the scaffolds at first (C1), fifth (C5), and tenth (C10) cycle of compression showing no statistically differences between cycles. (h) Representative strain-stress curves of compression experiments. BMSC viability after 72 h of culture on PEU 2D films (i) and cell surface density after 24 h (j). Fluorescence microscopy images of BMSCs after culture on PEU and TCP (tissue culture plate, polystyrene) (k). Attachment shows cells adhered to the different material substrates and stained for F-actin (Phalloidin, green) and DNA (Hoechst, blue). Alive and dead panels show cells simultaneously stained with calcein (green, alive) and ethidium bromide homodimer (red, dead). Scale bar is 200 μm. (For interpretation of the references to colour in this figure legend, the reader is referred to the Web version of this article.) BMSC, bone marrow–derived stem cell, PEU, poly(ester)urethane.

The scaffolds produced and tested in this study were fabricated with a BioScaffolder system (SysEng) equipped with a temperature controller and a G22 needle (400 μm internal diameter). PEU was deposited with a pore size and shape (angle of deposition) of 500 μm and 90° and 500 μm and 60° (Fig. 1). The polymer deposition was driven at a feed (travel) rate of 350 mm/R, a dispensing speed of 30 RPM, dispensing pressure of 0.8 MPa, and with a layer thickness of 0.3 mm. The optimal printing temperature to avoid degradation of the polymer was found to be 175 °C [41].

**Fig. 2:**
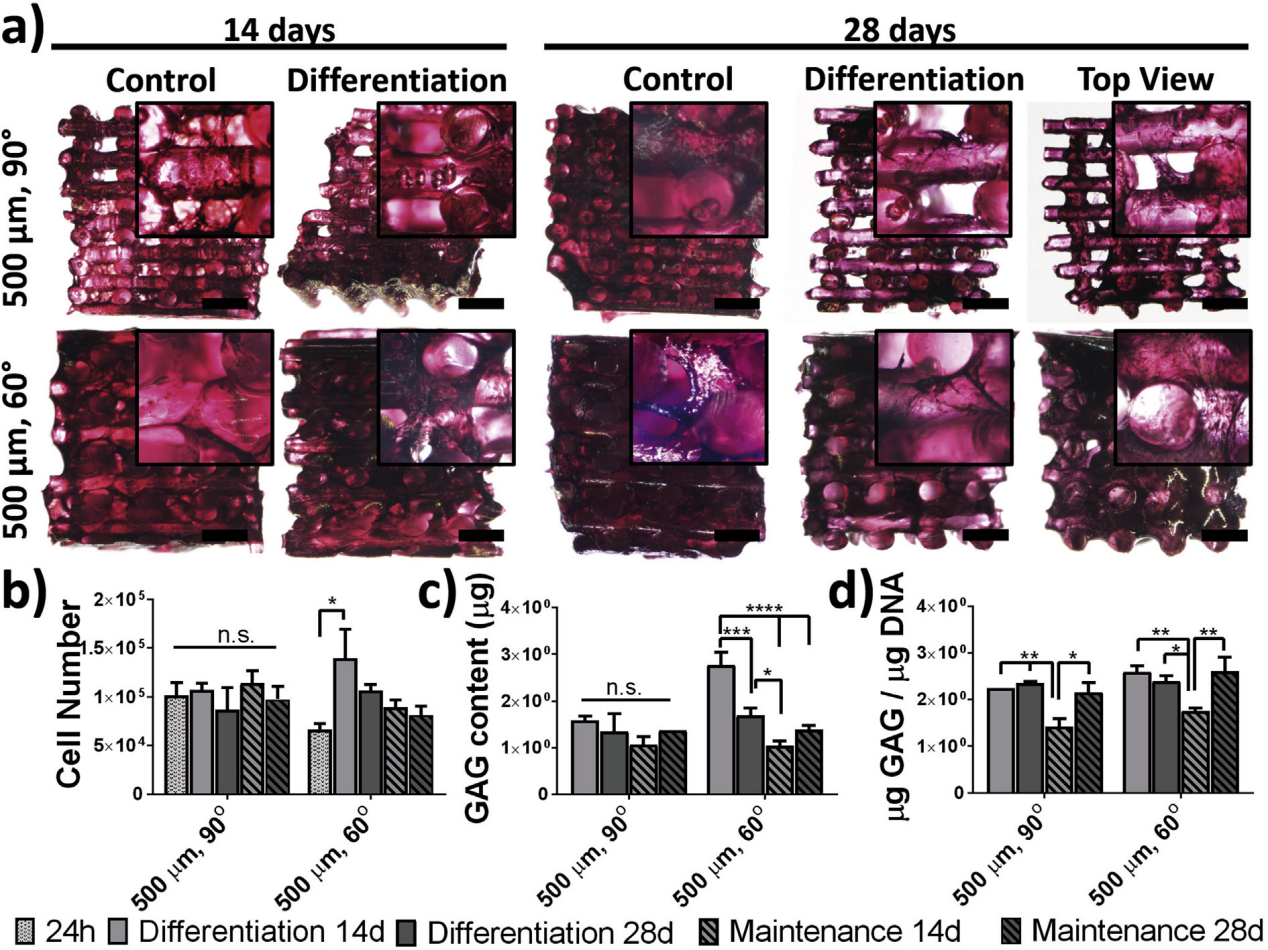
Glycosaminoglycan (GAG) deposition and cell proliferation. (a) Optical microscopy images of the cross-section and top views of scaffolds cultured for 14 and 28 days in basal (control) or differentiation media (differentiation) and stained with Safranin-O (GAGs, dark pink) and counter stained with Weigert's iron hematoxylin (black, cell nuclei). Scale bar is 1 mm. (b) Cell number populating the scaffolds after 24 h of seeding and 14 and 28 days of culture in either basal (maintenance) or differentiation medium. (c) Total GAG content and (d) DNA normalized GAG deposition on scaffolds cultured for 14 and 28 days in the maintenance or differentiation medium. (For interpretation of the references to colour in this figure legend, the reader is referred to the Web version of this article.)

### 2.3 Spin coating of 2D PEU films

Glass coverslips of 12 mm diameter (Fisher Scientific) were cleaned by placing them in 50 mL centrifugation tubes and consequently submerging the slides in isopropanol. The centrifugation tubes were then placed on a Branson 2510 ultrasonic cleaner and sonicated for 15 min. The isopropanol was then removed, and the coverslips were rinsed two times more in clean isopropanol. PEU was dissolved at a concentration of 20 mg/mL in CHCl_3_ (Sigma-Aldrich) and spin coated on top of precleaned glass coverslips. Spin coating was performed using a homemade instrument in 2 steps. In the first step, the PEU solution was added dropwise over a time of 30 s with a slide rotation speed of 1500 rpm. After addition of the polymer, the rotation speed was increased to 3000 rpm which allowed for film formation and CHCl_3_ evaporation.

### 2.4 Cell culture

Human BMSCs were isolated from a 22-year-old male donor by aspiration by Texas A&M Health Science Center after ethical approval from the local and national authorities and written consent from the donor [45]. Mononuclear cells were isolated by centrifugation, and isolated BMSCs were verified for differentiation capacity. Cryopreserved vials at passage 2 were plated at a density of 1000 cells/cm^2^ and cultured in alpha MEM (minimum essential media) supplemented with GlutaMax (Gibco, Massachusetts, USA) and 10% fetal bovine serum (FBS) (Sigma-Aldrich, Missouri, USA) (basal or maintenance media). Cells were subcultured at 80% confluence.

### 2.5 Cell culture on 2D substrates

PEU spin-coated glass coverslips were placed on a 24-well plate and sterilized by submerging them in 70% ethanol for 15 min. Afterward, samples were washed three times with Dubelcco's phosphate buffered saline (PBS, Gibco). After the last wash, BMSCs were seeded at a density of 5000 cell/cm^2^ on a volume of approximately 25 μL of α-MEM supplemented with GlutaMax (no FBS was used). After 24 h, samples were placed in new 24-well plates. For cell attachment experiments, samples were rinsed with PBS and fixed with 4% paraformaldehyde for 15 min, only after 24 h of culture. Samples were rinsed with PBS and permeabilized for 10 min in a 0.1% Triton X 100 solution in PBS. Samples were stained for F-actin and DNA with Phalloidin Alexa Fluor-488 (1:100, 1 h) and Hoechst (1:2000, 10 min), respectively. For cell viability studies, medium was replaced after 24 h with fresh α-MEM supplemented with GlutaMax and 10% FBS. After 72 h of culture, cells were rinsed with PBS and a live/dead viability kit consisting of ethidium homodimer and calcein (Thermo Fisher) was used to stain cells following the manufacturer's indications. After 30 min of incubation, cells were imaged using a Nikon TI-E epifluorescent microscope and analyzed using FiJi and CellProfiler free software.

### 2.6 Cell culture in FDM scaffolds

#### 2.6.1 Cell seeding

Scaffolds of PEU with a 500 μm pore size and a pore angle of 90° and 60° were cut using a biopsy puncher to obtain cylindrical samples of 4 mm diameter and 4 mm height. Samples were distributed on 50 mL centrifugation tubes and sterilized by immersing them in a 70% ethanol solution for 15 min, after which samples were washed thoroughly with PBS three times. To promote initial cell attachment, samples were coated with truncated recombinant human vitronectin (Thermo Fisher), a protein commonly used for the culture of pluripotent stem cells. Vitronectin coats the scaffolds in a non-specific manner and is washed away after media changes. Vitronectin coating was performed at a concentration of 1 μg/cm^2^, assuming a surface area per scaffold of 16 mm^2^. In brief, vitronectin stock solution was diluted in PBS to reach a concentration of 1 μg/mL^1^. The scaffolds were distributed in 15 mL falcon tubes and covered with the vitronectin solution at a ratio of 6 scaffolds per each 1 mL of solution. The coating was left to proceed at room temperature (RT) for 1 h in an orbital shaker. Afterward, samples were placed on 24-well plates, and 1.8·10^5^ cells per scaffolds (3.6·10^6^ cell/cm^3^) were seeded at a concentration of 3.6·10^6^ cell/mL (that is 50 μL of cell dispersion per scaffold) without previous scaffold rinsing. Cells were left to attach to the scaffolds for 2 h in the incubator (37 °C, 5% CO_2_). Thereafter, the scaffolds were flipped and left to incubate for another 2 h. After 4 h of initial cell seeding, 1.5 mL of basic medium supplemented with penicillin (100 units/mL) and streptomycin (100 μg/mL) was added to each well (1 scaffold per well).

#### 2.6.2 Cell differentiation

The samples were cultured for 7 days in basal media. After this period, the differentiation process was started, when relevant. Samples for differentiation studies were cultured in chondrogenic media consisting of high glucose (4.5 mg/mL) DMEM (Dubelcco`s Modified Eagle Medium) with 100 μg/mL of sodium pyruvate (Gibco), 0.2 mM l-ascorbic acid-2-phosphate (Sigma-Aldrich), 1% 100x ITS (insulin-transferrin-selenium) liquid media supplement (Thermo Fisher Scientific), 40 μg/mL proline (Sigma-Aldrich), 100 U/mL penicillin/streptomycin, and 100 nM dexamethasone (Sigma-Aldrich). To the complete media and right before addition to the cultures, 0.01ug/mL of transforming growth factor-β3 was supplemented (Peprotech). Media for both maintenance and differentiation conditions were changed every second day.

### 2.7 Cell pellet culture

BMSCs were cultured on pellets as a positive control of the experiment because this method is a ‘gold standard’ for in vitro chondrogenesis of stem cells. To form pellets, 0.25·10^6^ cells were placed in a 15 mL polypropylene tube with 500 μL maintenance media and centrifuged at 500 rcf for 5 min to form a flat pellet at the bottom of the tube. The tubes were left (covered with the cap but allowing gas transfer) in the incubator for 24 h after which rounded pellets were spontaneously formed. After 24h of seeding, media were replaced for differentiation or maintained in basic media conditions (control of the control pellets). Media change was performed every second day, as for the scaffolds.

### 2.8 DNA assay

For DNA assays, samples were harvested and placed in Eppendorf tubes. Samples were first frozen at −80 °C and then thawed at room temperature (RT), this process was repeated 3 times. The extracellular matrix was digested by a proteinase K treatment. In brief, samples were placed in 1.5 mL Eppendorf tubes and incubated overnight at 56 °C with 250 μL of 50 mM Tris/1 mM ethylenediaminetetraacetic acid/1 mM iodoacetamide solution containing 1 mg/mL^1^ proteinase K and 10 μg/mL pepstatin A. After sample digestion, the samples were freeze-thawed 3 times in liquid N_2_ to facilitate the DNA extraction. DNA was measured with a CyQuant cell proliferation assay kit (Thermo Fisher). In brief, cellular RNA was degraded by incubating the samples for 1 h at RT with lysis buffer containing RNase A. 100 μL of each sample (triplicates) were placed in a 96 well plate, and 100 μL of 2x GR-dye solution were added and left to incubate for 15 min at RT. A standard curve was prepared with a DNA standard solution, and fluorescence intensity was measured at 520 nm.

### 2.9 Glycosaminoglycan assay

Samples from proteinase K digestion step (DNA assay) were used to measure glycosaminoglycan (GAG) content using a 1,9-dimethyl-methylene blue zinc chloride double salt ([DMMB], Sigma-Aldrich) solution (16 mg DMMB in 5 mL ethanol). In brief, 150 μL of DMMB solution was mixed with 25 μL of the sample and 5 μL of 2.3 M NaCl on a black well plate; the absorbance difference at 525 and 595 nm was measured. Data were compared with a standard curve prepared with chondroitin sulfate from shark cartilage (Sigma-Aldrich).

### 2.10 Histology

Scaffold-tissue constructs for histological analysis were cut opened along the height axis. Samples stained for GAGs were first stained for 10 min in Weigert's iron hematoxylin solution (Sigma-Aldrich) to counterstain the cell nucleus black, washed thoroughly in running tap water and stained for 3 min with Fast green solution (Sigma-Aldrich), and quickly dipped in 0.5% acetic acid. Finally, samples were stained for 5 min in a 0.1% solution of Safranin-O (Sigma-Aldrich), after which they were rinsed with PBS until the solution was clear. Picrosirius red (Abcam) staining was performed following the manufactures’ instructions. In brief, samples were incubated for 2 h in Picrosirius red solution and then rinsed quickly in 0.5% acetic acid solution (both from Abcam, Cambridge, UK). Afterward, samples were rinsed in PBS 3 times.

### 2.11 Immunofluorescence

Samples were cut opened by half along the longitudinal axis with a scalpel and prepared by fixing them in 4% paraformaldehyde for 30 min, followed by rinsing in PBS and permeabilization for 15 min in a 0.1% Triton X-100 solution (Millipore Sigma) in PBS. Blocking was performed for 1 h in a solution of 3% bovine serum albumin (BSA, Sigma-Aldrich) and 0.01% Triton X-100 in PBS. After rinsing the blocking solution, samples were incubated for 1 h at RT with mouse anti-collagen I (1:500) (ab23446, Abcam), rabbit anti-collagen II (1:400) (ab34712, Abcam) antibodies, and AlexaFluor 564 Phalloidin (1:100) (Thermo Fisher Scientific). After 1 h, incubation was stopped and the solution was rinsed 3 times with 0.3% BSA and 0.01% Triton X-100 in PBS. Secondary antibodies, anti-rabbit AlexaFluor 647, and anti-mouse AlexaFluor-488 (both from Thermo Fisher Scientific) were incubated in the dark for 30 min at RT in PBS (1:200), followed by rinsing with PBS and staining with Hoechst 33342 (1 mg/mL, 1:3000) (Thermo Fisher Scientific) in PBS for 10 min. After final rinsing with PBS, samples were observed under a Leica TCS SP8 CARS confocal microscope.

### 2.12 Gene expression

Total RNA was extracted with the RNeasy Minikit with on column DNase treatment (Qiagen, Hilden, Germany) according to the manufacturer's protocol. cDNA was synthesized from 200 ng total RNA, using iScript cDNA synthesis kit (Bio-Rad, California, USA) on a 20 μL reaction following manufactures instructions. Real-time polymerasa chain reactions (RT-PCRs) were prepared on a total volume of 20 μL with 10 μL iQ SYBR green Supermix (Bio-Rad), 0.2 μM forward and reverse primers (Table 1), 3 ng of cDNA and diethylpyrocarbonate (DEPC)-treated water. For no RT controls, an equivalent volume of DNase- and RNase-free water was used. A CFX96™ IVD Real-Time PCR system (Bio-Rad) was used with a thermal cycle of 50 °C for 2 min, 95 °C for 2 min, and then 95 °C for 15 s and 60 °C for 30 s for a total of 40 cycles. Ct values of RT-PCR were normalized against the house-keeping gene and analyzed using the ΔΔCt model [46].

**Table 1:** List of primers used for RT-PCR experiments.

Gene	Forward primer 5′ to 3′	Reverse primer 5′ to 3′
GAPDH	ATG GGG AAG GTG AAG GTC G	TAA AAG CAG CCC TGG TGA CC
Sox9	TTC CGC GAC GTG GAC AT	TCA AAC TCG TTG ACA TCG AAG GT
Sox5	ATAAAGCGTCCAATGAATGCCT	GCGAGATCCCAATATCTTGCTG
Sox6	GGATGCAATGACCCAGGATTT	TGAATGGTACTGACAAGTGTTGG
Col1a1	AGGGCCAAGACGAAGACATC	AGATCACGTCATCGCACAACA
Col2a1	GGCAATAGCAGGTTCACGTACA	CGATAACAGTCTTGCCCCACTT
Col Xa1	GAC TCC CTC CTC ACT GTC GC	AGG GAA GTC TCC CTC ACT TGT
RunX2	AGT GAT TTA GGG CGC ATT CCT	GGA GGG CCG TGG GTT CT

### 2.13 Mechanical testing

The mechanical properties of the scaffolds were measured before and after culture. Bare scaffolds were tested in air and cell-scaffold constructs in PBS. Samples were prepared as cylinders of 4 mm diameter and 4 mm height. Before the tests, samples were measured with a caliper, and the dimensions were noted down for data analysis. The compressive mechanical properties of the bare scaffolds (dry) with different architectures and of the scaffold-tissue constructs after different cell culture time (in PBS) were measured on a TA ElectroForce system (TA Instruments) equipped with a 450 N load cell under unconfined and non-equilibrium conditions. The instrument was controlled with Wint7 software. Tests were conducted at a strain rate of 0.01 mm/s. The experiments were run until approximately 50% deformation (or until the maximum applicable load was reached). The Young's modulus was calculated from the slope of the strain-stress curve between 0.2% and 1.2% engineering strain. The yield stress and strain were calculated from the stress-strain curves as the maximum value of the elastic regime before the inflexion point. Cyclic compression experiments were performed on scaffolds cylinders of 6 mm diameter and 4 mm height that were also first measured with a caliper. Tests were conducted up to 10 cycles at a frequency of 0.01 Hz (strain rate of 0,008 mm/s^1^) with a 45 N load cell. The experiments were run until approximately 10–12% deformation (fixed deformation of 0.4 mm).

### 2.14 Micro computed tomography

Micro computed tomography (μCT) scans were recorded with a Bruker Skyscan 1272 11Mp scanner with cone beam geometry, equipped with a 4032 × 2688 detector. The scanner was air damped to reduced vibration disturbances. Alignment, thermal drift of the cathode spot, beam hardening, and ring artefacts were corrected using Bruker's software. The isotropic voxel size was 3^3^ μm^3^. The obtained 3D data sets were reconstructed using FDK implemented in NRecon 1.7.1.0 (Bruker microCT) and analyzed with CTAN software (Bruker) [47]. An object volume (VOI) corresponding to the whole scaffold was selected to balance potential morphological differences due to printing. The VOI selection was refined to take into account the scaffold morphology resulting from the deposition pattern used, including an integer amount of repeating cells. Scaffold parameters such as porosity, Euler number, volume, and surface area were calculated via the 3D analysis function available in the software.

### 2.15 Statistical analysis

Statistical significance was calculated for Young's modulus measurements, DNA assay, GAG assay, and PCR analysis by two-way analysis of variance (ANOVA) with Tukey's multiple comparison test: (∗∗∗∗) *p* < 0.0001, (∗∗∗) *p* < 0.001, (∗∗) *p* < 0.01, and (∗) *p* < 0.1.

## 3 Results

### 3.1 Fabrication and structural characterization of PEU scaffolds

**Fig. 3:**
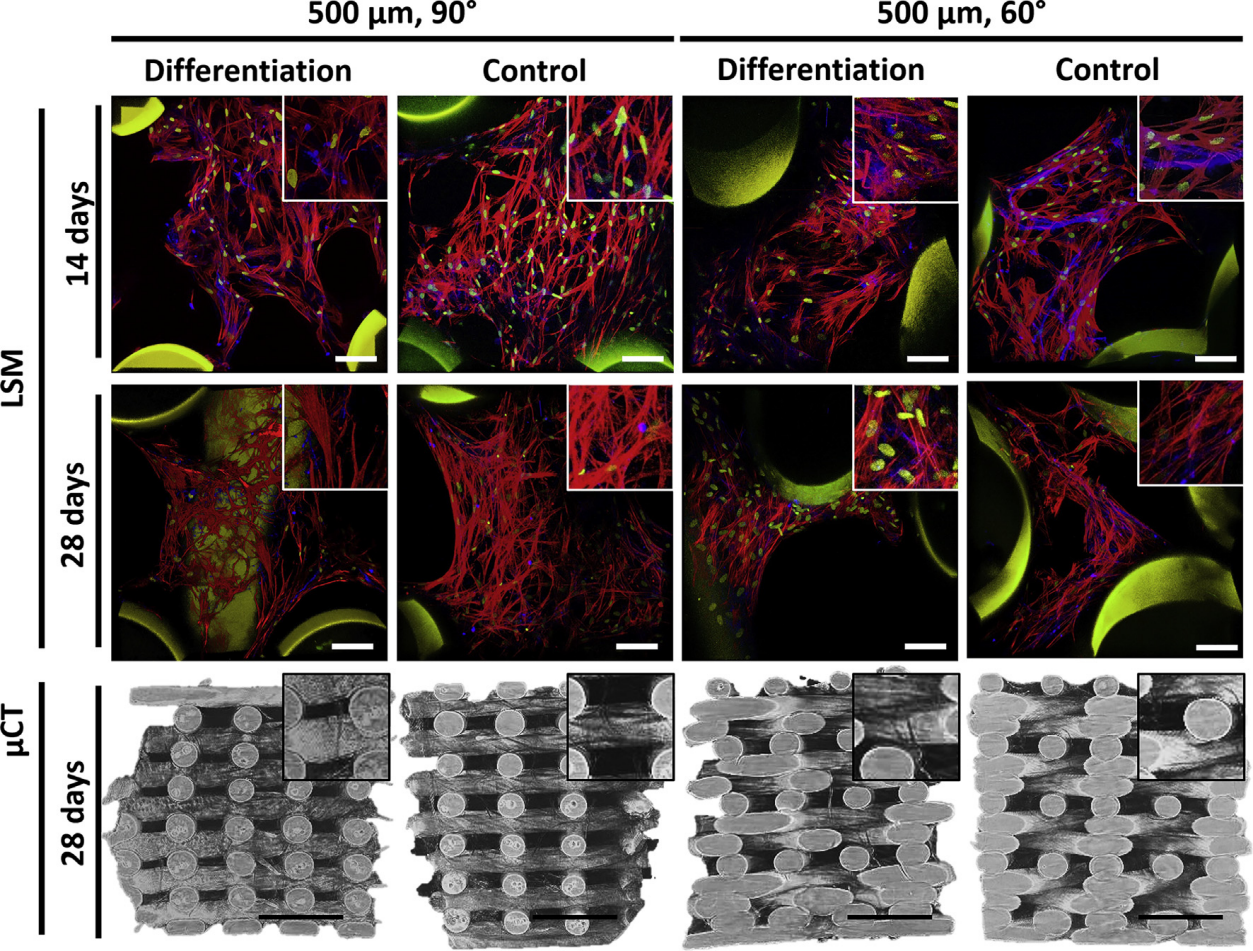
Extracellular matrix deposition. Laser scanning microscopy (LSM) images (top rows) of BMSCs cultured for 14 and 28 days in basal (control) and differentiation medium on scaffolds with 500 μm and 90° or 60° deposition patterns. BMSCs were immunostained for F-actin (Phalloidin, red), nucleus (Hoechst, yellow), collagen I (green), and collagen II (blue). Scale bar is 100 μm. μCT images (bottom row) of PEU scaffolds after 28 days of culture in differentiation and basal medium showing ECM deposition. Scale bar is 1 mm and insets are 700 × 700 μm. (For interpretation of the references to colour in this figure legend, the reader is referred to the Web version of this article.) BMSCs, bone marrow–derived stem cells; μCT, micro computed tomography; PEU, poly(ester)urethane.

To assess the functionality of additive manufactured PEUs for the regeneration of cartilage with BMSCs, scaffolds with pore size and shapes of 500 μm and 90° or 60° were fabricated. The structure of the FDM scaffolds was analyzed by scanning electron microscopy (SEM) (Fig. 1a–d), showing high fidelity with the designed patterns, as previously reported [41]. Analysis of the scaffolds by μCT revealed porosities of 57 ± 3% and 52 ± 3% for the 90° and 60° deposition patterns, respectively (Table 2 and Fig. 3). A slightly higher closed porosity (a connected assemblage of space (black) voxels that is fully surrounded on all sides in 3D by solid (white) voxels) was detected for the 60° patterned scaffolds (2.9 ± 0.4% vs 0.19 ± 0.06%), increasing the actual open porosity difference between deposition patterns. Consequently, also higher total surface area and pore volume were measured for scaffolds with 90° patterns. In contrast to these data, and as reported previously, the scaffolds with a 60° pattern presented a higher connectivity (Euler number, lowest tortuosity) than that with 90° patterns.

**Table 2:** Porosity, closed porosity, total surface area, total available pore volume and connectivity of fabricated scaffolds (assuming a total volume of 50 mm^3^).

Scaffold pore size and pattern angle	Porosity (%)	Closed porosity (%)	Total surface area (mm^2^)	Total pore volume (mm^3^)	Connectivity (Euler number)
500 μm, 90°	57 ± 3	0.19 ± 0.06	549 ± 4	28 ± 1	13834 ± 1777
500 μm, 60°	52 ± 3	2.9 ± 0.4	379 ± 20	26 ± 1	6998 ± 2828

### 3.2 Mechanical properties of additive manufactured scaffolds

The fabricated PEU scaffolds were analyzed by mechanical compression, and representative stress-strain curves are shown in Fig. 1, h. The stress-strain traces of the two scaffold architectures present the typical response of porous materials with an initial elastic regime that is followed by the collapse of the pores. This usually appears as a plateau in the curve that in this case is more representative of the 60° patterned scaffold. On the 90° pattern, however, this area appears as a first densification regime, probably due to the lower thickness of the pores (2 layers on 90° vs 3 layers on the 60° pattern). After the collapse of the pores, the structure undergoes the so-called densification step, characterized by an exponential increase of the mechanical properties. The Young's modulus was calculated from the initial elastic regime of these curves, resulting on 4.2 ± 0.9 and 3.1 ± 0.8 MPa for 90° and 60° patterns, respectively (Fig. 1g–h). As expected, the deposition pattern influenced the Young's modulus of the scaffolds. The yield strain (that corresponding to the yield strength) was of approximately 25% for 90° patterns and 35% for 60° patterns. While this extended yield strain is common to PEUs, it is high as compared with traditionally FDM scaffolds of other biopolymers [29,30]. Cyclic compression experiments with a maximum strain of 10–12% (fixed deformation of 0.4 mm) were performed to test the resilience of the scaffolds (Fig. 1e–f). The samples showed an outstanding shape recovery capability with maximum deformations after 10 cycles, less than 0.5% and 1% for 90° and 60° patterns, respectively (Fig. S1). The Young's modulus of the scaffolds remained invariable over the compression cycles (Fig. 1, g).

### 3.3 BMSC viability and attachment on PEU films

Before the investigation of the potential of PEUs as cartilage tissue engineering scaffolds, the cell viability and capability to adhere to PEU films was evaluated with BMSCs. Cells attached to PEU films and to the common polystyrene tissue culture plate (TCP) presented a similar morphology with well spread cell bodies (Fig. 1, k). PEU films presented a higher number of attached cells than TCP after 24 h of seeding (Fig. 1, j), with cell densities of 4.3^.^10^3^ ± 0.5 and 3.2^.^10^3^ ± 0.7 cell/cm^2^, respectively, probably due to slight differences on the hydrophilicity of the two substrates. Cell viability was visualized after 72 h of cell seeding showing a high amount of viable cells (Fig. 1k, alive) and very few dead cells (Fig. 1k, dead) for both cells cultured on PEU films and TCP. Cell viability was quantified from the images and values of 82 ± 4 and 96 ± 2% were measured for PEU and TCP, respectively (Fig. 1, i).

### 3.4 In vitro cartilage formation

The capability of PEU additive manufactured scaffolds to drive the differentiation of BMSCs was tested in vitro. BMSCs were cultured for 14 and 28 days in differentiation media on PEU scaffolds with different pore architectures, namely with a 500 μm pore size and 90° or 60° pattern (Fig. 1, a-d). As comparison, BMSCs were also cultured on the same scaffolds in basal media and as pellets in both differentiation and basal (maintenance) conditions.

#### 3.4.1 Cell proliferation in additive manufactured PEU scaffolds

After 14 and 28 days of BMSC culture in the PEU scaffolds in differentiation and maintenance media, the total DNA content was measured (Fig. 2, b). For comparison, samples after only 24 h of culture were also analyzed. The seeding efficiency on PEU scaffolds precoated with truncated human vitronectin was of approximately 50% for 90° and 30% for 60° patterns. Although these values denote a low seeding efficiency, these are high as compared with non-coated scaffolds (50% and 30% efficiency vs. 10–20% for both 90° and 60° patterns), which is in line with other biomaterials used for additive manufacturing and seeded with BMSCs (Fig. S2)[48,49]. Thus, values of 102·10^3^ ± 22·10^3^ and 66·10^3^ ± 10·10^3^ cells per scaffolds were quantified after 24 h of seeding for the 90° and 60° patterns, respectively (Fig. 2, b). Although a higher tortuosity was calculated for 90° patterned scaffolds, the surface area was also higher (549 ± 4 vs 379 ± 20) (Table 2), which explains this difference on initial cell attachment. Indeed, the cell density (cell number/surface area) on the scaffolds is similar for both printing patterns, and the cell surface density (cell number/pore volume) appears to be higher for scaffolds with a 60° pattern (Figure S3, a and b). After 14 and 28 days of culture, the cell number remained constant (not significantly different) for scaffolds with a 90° pattern and slightly increased in 60° scaffolds, reaching after 28 days of culture values of 106·10^3^ ± 10·10^3^ and 81·10^3^ ± 9·10^3^ cells for samples in differentiation and maintenance media, respectively. No significant differences were found between samples cultured in differentiation and basal medium. This effect is commonly observed when culturing BMSCs in additive manufactured scaffolds [48]. The same observation was done on control pellet cultures in maintenance and differentiation medium (Fig. S4).

#### 3.4.2 Deposition of specialized extracellular matrix

Articular cartilage has a characteristic extracellular matrix (ECM) composition that is rich in GAGs and collagen II [50,51]. Thus, it is important to verify that the composition of the engineered neotissue resembles that one of the native one. The deposition of GAGs during the differentiation process was evaluated after 14 and 28 days of culture via colorimetric quantification from the digested samples and via histological (Safranin-O) staining. Histological Safranin-O staining and observation of PEU scaffolds after 14 and 28 days of culture showed a clear deposition of GAGs (red stain) (Fig. 2, a). The GAG deposition and distribution of cells was homogenous with similar amounts on the inside and outside of the scaffolds (Fig. 2, a, top view). The deposition of GAGs appeared to be similar on differentiation samples as compared with controls, highlighting the potential of the materials to promote the deposition of a cartilaginous matrix. The pores of the scaffolds with a 60° pattern presented an apparent higher amount of cells and GAG, particularly on differentiation samples after both 14 and 28 days of culture. Contrary, scaffolds with a 90° pattern appeared to have open pores, even after 28 days of culture in both media conditions. This visual observation supports our findings on cell density (Fig. S3). The deposition of GAGs was also analyzed quantitatively by the DMMB assay (Fig. 2c and d) for all scaffolds on the different structural designs. The deposition of GAGs on the scaffolds was overall high with total GAG values that range from 1 to 2.7 μg after 14 days of culture and 1.3–1.7 μg after 28 days of culture. A decreased GAG deposition was measured for all the samples at longer culture times, which could be an indication of ECM remodeling. The deposition after 14 days was higher in differentiation than in maintenance conditions for both pore structures (Fig. 2c and d), becoming significant upon normalization to the total DNA amount (2.24 ± 0.02 vs. 1.4 ± 0.3 μg GAG/μg DNA for 90° patterns and 2.6 ± 0.2 vs. 1.75 ± 0.09 μg GAG/μg DNA for 60° patterns) (Fig. 2, d). This difference in GAG deposition was, however, compensated after 28 days of culture where values in differentiation and maintenance conditions were not significantly different (2.34 ± 0.06 vs. 2.2 ± 0.3 μg GAG/μg DNA for 90° patterns and 2.4 ± 0.2 vs. 2.6 ± 0.5 μg GAG/μg DNA for 60° patterns). Comparison between the density of GAG deposition (GAG/pore volume) and GAG surface density (GAG/surface area) between scaffolds with different printing patterns showed that 60° scaffolds present a significantly higher amount of GAGs after 14 and 28 days of culture in differentiation media than their counterparts (Figure S5 a and b). These results suggest an optimal cell differentiation and ECM deposition on scaffolds with this deposition pattern. As a control, the amount of GAGs deposited by BMSCs cultured on pellets (gold standard for chondrogenesis in vitro) showed, after 28 days, values of 0.6 and 0.2 μg of GAG per μg of DNA for cells in differentiation and culture media, respectively (Fig. S6). These values are 4- and 8-fold lower, than what were measured on the PEU scaffolds. Moreover, the high values on GAG deposition in both 90° and 60° patterned scaffolds, together with the similar depositions measured for the differentiation and control conditions after 28 days of culture, suggest that PEU scaffolds have an intrinsic chondrogenic potential.

The cartilage accounts for a specialized extracellular matrix with a high percentage of collagen II. Evaluation of the total collagen being deposited on the FDM scaffolds was performed qualitatively via histology and the presence of specific collagens (collagen type II and I) via immunofluorescence. Histological evaluation of collagen deposition was performed using Picrosirius red staining, a dye that stains all present collagen types in red/orange. The deposition of collagens on PEU scaffolds was overall high (Fig. S7). Collagen (red stain) was visible on the entire sample with more intense staining on the cell bodies. A comparison between samples cultured for 14 or 28 days showed an increase in collagen deposition over time that was most pronounced in control samples than in differentiation ones. Moreover, comparison between these controls and differentiation samples showed no apparent difference in collagen deposition. The deposition of collagen in the different structures suggests that a 60° pore shape is preferential for a chondrogenic differentiation with slightly higher amounts of collagen being deposited (more intense stain).

The deposition of specific collagens was evaluated via immunofluorescence. BMSCs on pellet cultures showed a high extracellular deposition of collagen II and only intracellular (nuclear) expression of collagen I (Fig. S8). PEU scaffolds in differentiation and control conditions after 14 and 28 days of culture were stained for collagen II (blue) and collagen I (green), F-actin (red), and DNA (yellow) (Fig. 3). Scaffolds with a 90° pattern showed intracellular expression of collagen II in both control and differentiation samples already after 14 days of culture, which became more pronounced after 28 days. Scaffolds with a 60° pattern showed deposition of collagen II in both control and maintenance conditions after only 14 days of culture and later (28 days), indicating that the structure of the scaffolds could be favoring the chondrogenic differentiation of the cells. The cells within the scaffolds also presented a rather elongated morphology with cell-cell interactions that are not characteristic of chondrocytes in the hyaline cartilage.

#### 3.4.3 Cell invasion and formation of homogeneous tissue

To study the overall cell invasion and tissue formation within the PEU scaffolds, μCT scans were recorded. Characteristic images of the 3D reconstructions are shown in Fig. 3. Scaffolds printed with a 500 μm and 90° pattern showed a great cell invasion and deposition of ECM as visualized by the darker fibrous areas present on the core of the scaffold. Samples in differentiation media seemed to have a higher cell density or ECM deposition than their counterparts in maintenance media. Scaffolds with 60° deposition pattern also showed a high cell/ECM density that again seemed to be higher on the differentiation condition than that in the control sample. It is noteworthy that the formation of sheet-like structures bridging the fibers between pores is probably the result of cell aggregation and ECM deposition, indicative of tissue formation.

#### 3.4.4 Gene expression profile

**Fig. 4:**
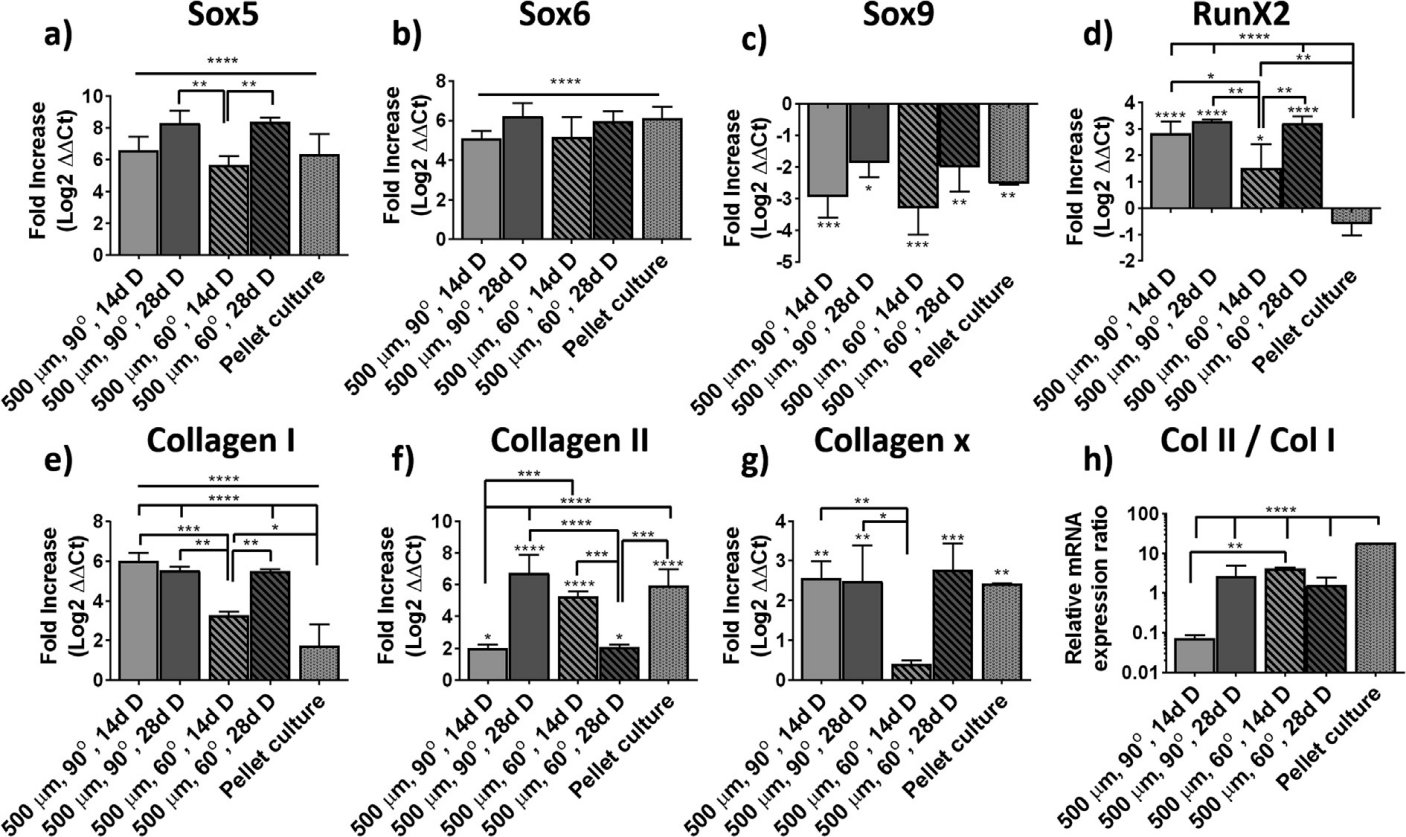
Gene expression analysis. Fold increase (a–g) of Sox5, Sox6, Sox9, RunX2, collagen I, collagen II, and collagen X gene expression of BMSCs after 14 and 28 days cultured in 500 μm and 90° or 60° patterned scaffolds in differentiation media. (h) Collagen II to collagen I relative mRNA expression ratio of the same samples. BMSC, bone marrow–derived stem cell.

To characterize the differentiation state of the cells, their relative gene expression was analyzed, initially before the experiment and after 14 and 28 days of differentiation (Fig. S9, a, and Fig. 4). The gene expression profile of cells cultured following the gold standard pellet culture was also analyzed for comparison (Fig. S9, b, and Fig. 4). BMSCs before culture in PEU scaffolds showed the characteristic phenotype after in vitro expansion, with relatively high expression of collagen I, Sox9, and RunX2. The expression of collagen II and X was low, as well as the expression of Sox5 and Sox6 (Figs. S9 and a). Upon pellet culture in chondrogenic media, there was a 6-fold upregulation of both, Sox6 and Sox5, reaching similar expression values to that of Sox9 (Figs. S9 and a). Collagen II was also upregulated approximately 6-fold, much higher than collagen I. The gene expression of collagen I was also upregulated, although this was within the 2-fold increase range (generally considered as not significant).

**Fig. 5:**
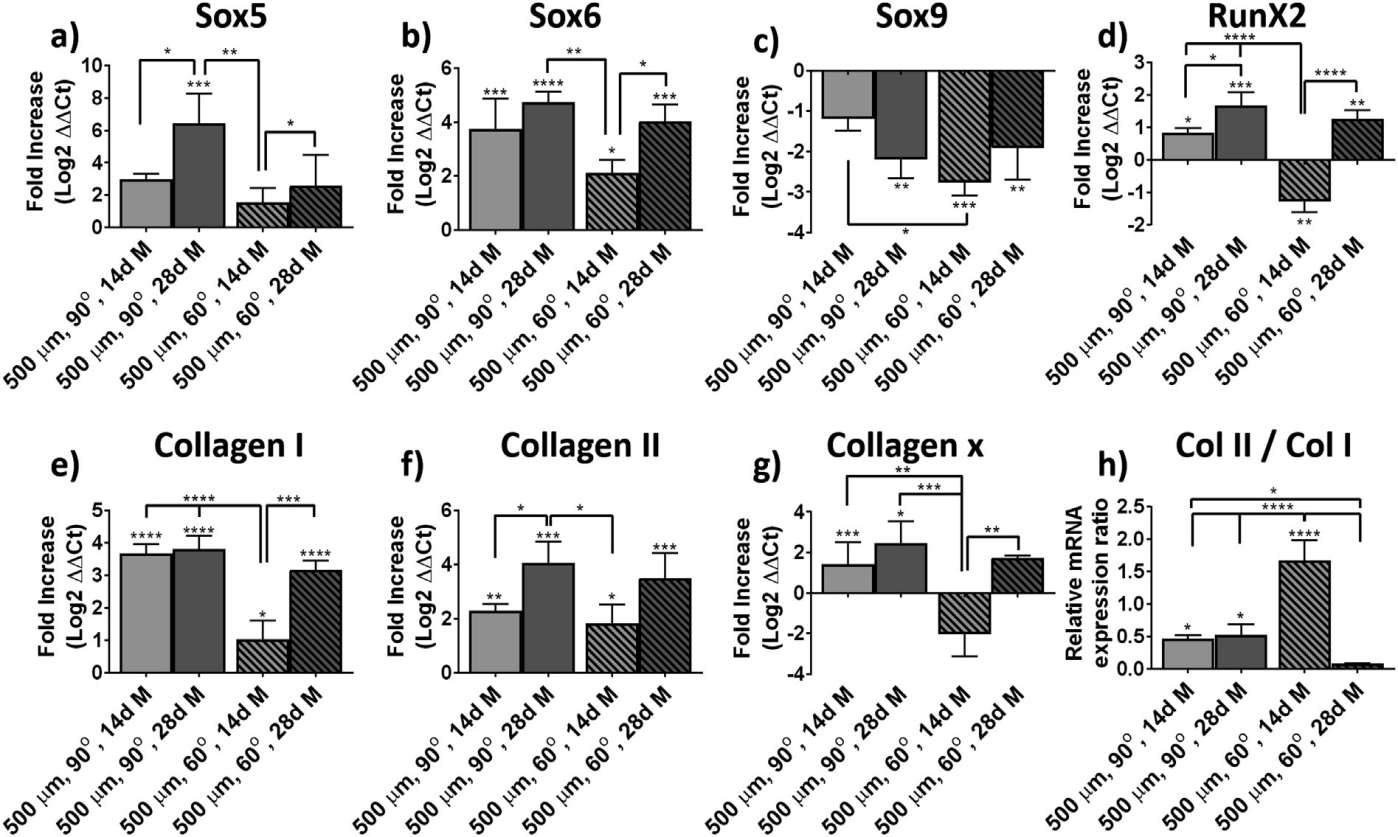
Fold increase of (a–g) Sox5, Sox6, Sox9, RunX2, collagen I, collagen II, and collagen X gene expression of hMSCs after 14 and 28 days cultured in 500 μm and 90° or 60° patterned scaffolds in basal media. (h) Collagen II to collagen I relative mRNA expression ratio of the same samples.

The gene expression of cells cultured on PEU scaffolds after 28 days showed a similar profile of that of the gold standard pellet culture (Fig. 4). The gene expression of Sox5 was approximately 6-fold upregulated for both 90° and 60° scaffolds after 14 days. This upregulation increased after 28 days of culture to 8-fold for both scaffold architectures. The expression of Sox6 was upregulated by 5- and 6-fold after 14 and 28 days of culture for both scaffold types. Sox9 appeared downregulated by 3- and 2-fold after 14 and 28 days of culture, respectively, which was similar to pellet cultures. The deposition of collagen I was also upregulated, showing a higher fold increase than that for the pellet cultures and reaching values of approximately 6-fold after 28 days of culture. The higher expression of collagen I can be explained as a consequence of the differentiation media used, containing ascorbate-2-phosphate and known to stimulate the production of all collagens. Thus, this upregulation is less pronounced in cells cultured on scaffolds in basal media conditions (Fig. 5). The expression of collagen II was different for scaffolds with 90° and 60° patterns, showing an increase over time for the former and a decrease for the latter. These data confirmed what was observed by immunofluorescence. That is, a lower deposition of collagen II was found on the 90° patterns but the increased expression after 28 days may potentially have resulted on a later deposition of the protein if the scaffolds would have been left in culture for a longer period of time. Contrary, scaffolds with a 60° pattern showed a higher expression of collagen II at 14 days than at 28. This could be correlated with the collagen II deposition that was observed already at day 14 by immunofluorescence. Collagen X and RunX2 appeared both to be upregulated after 14 and 28 days of culture for both scaffold architectures. In all the cases, the upregulation was lower than 4-fold.

Cells cultured on PEU scaffolds in maintenance media over 14 and 28 days showed a similar gene expression profile as cells cultured in differentiation media (Fig. 5). In this case, however, the fold increase was smaller as expected. In general, the upregulation of Sox5, Sox6, collagen I, and collagen II was 2-fold lower than that for cells in differentiation media. The expression of RunX2 and collagen X was also lower than that of cells cultured in differentiation media. Taken all together, we suggest that cells cultured in control conditions have a less chondrogenic phenotype but still a significant upregulation of chondrogenic markers that is particularly remarkable given that this is solely the effect of the material and the 3-dimensional culture.

#### 3.4.5 Mechanical properties of the tissue-scaffold constructs

**Fig. 6:**
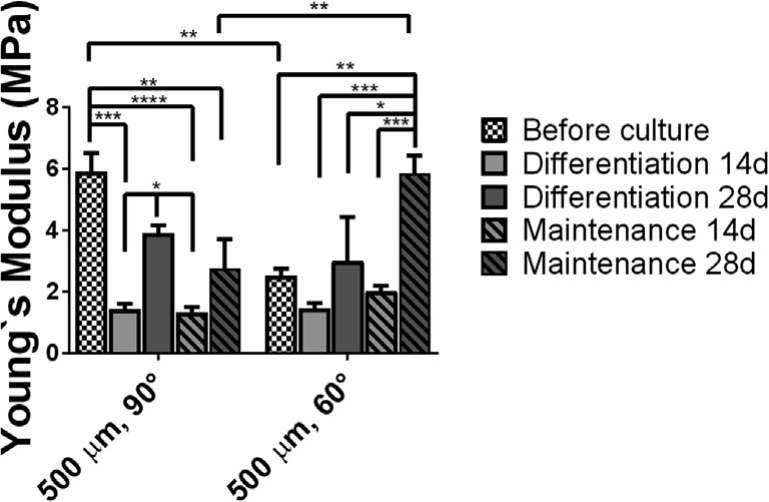
Compressive Young's modulus in PBS of cell-scaffold constructs before and after 14 and 28 days of culture in basal (maintenance) and differentiation media. PBS, phosphate buffered saline.

The Young's modulus (E′) under compression in liquid and non-equilibrium conditions was measured for the different scaffolds after 14 and 28 days of culture. The Young's modulus of PEU scaffolds showed increasing mechanical properties with longer culture periods, that is, generally ascribed to an increased amount of deposited ECM and tissue formation (Fig. 6). Comparison of samples cultured in differentiation and maintenance media supported the hypothesis that the material itself promotes the formation of neotissue. The mechanical properties of the scaffolds at 14 days showed similar Young's modulus for scaffolds with 90° and 60° deposition pattern with values of 1.5 ± 0.2 MPa and 1.5 ± 0.3 MPa, respectively, for scaffolds in differentiation media and 1.3 ± 0.3 MPa and 2.0 ± 0.3 MPa, respectively, in maintenance media. After 28 days of culture, scaffolds with 90° and 60° printing patterns displayed a Young's modulus of 3.9 ± 0.5 MPa and 3.0 ± 0.5 MPa, respectively, in differentiation media and of 2.8 ± 0.9 MPa and 5.8 ± 0.6 MPa, respectively, in maintenance media.

## 4 Discussion

Biodegradable PEUs, characterized by their elasticity and resilience, have emerged as potential scaffold materials to replace tissues that require high resilience and elasticity such as the heart and cartilage [36,52,53]. However, due to their viscoelastic properties and potential degradability when high temperatures are to be applied, their exploitation in FDM has only being recently reported by us. We reported on the fabrication of PEU scaffolds via additive manufacturing, which leads to elastic materials with a fabrication reproducibility (error) of 4 ± 1% and an average fiber diameter of 418 ± 5 μm, when a needle of 400 μm (inner diameter) was used. The additive manufacturing process resulted on an initial degradation step, with the subsequent decrease on molecular weight to approximately 80,000 kg/mol, which was then kept constant during 2 h of continuous deposition [41]. Following this same FDM process, scaffolds with a 500-μm fiber spacing and a deposition pattern of 90° and 60° were fabricated. Structural analysis of the scaffolds by SEM and μCT showed a reproducible and interconnected pore structure with a closed porosity lower than 3%. The calculated connectivity was higher for the smaller pore angle structure, which is in agreement with our previously reported data. A smaller surface area was also measured for these scaffolds than for that fabricated with a 90° angle.

Cartilage tissue engineering requires of the fabrication of scaffold materials that are initially capable of supporting the loads applied onto the knee until the new tissue is formed. The Young's modulus of human articular cartilage has been determined to be of 0.3 ˗ 0.8 MPa [54,55]. Although fabricating scaffolds capable of supporting such loads has been shown to be feasible, these are usually too stiff, resulting in low flexibility (or strain) with strain yields lower than 20%, typically lower than 5%, or strains that are not recovered upon cyclic load and thus, a mechanical mismatch between the native and the implanted tissue-scaffold construct [[56], [57], [58]]. Despite the higher Young's modulus measured for the scaffolds presented here (3.9 ± 0.5 MPa and 3.0 ± 0.5 MPa for 90 and 60° patterns, respectively, after 28 days of culture in differentiation media) as compared with the cartilage, degradation and resorption is expected to occur together with tissue formation, allowing for the restoration of the native properties of the tissue. Moreover, the knee undergoes repetitive loading during the day reaching, at the end of the day, strains of up to 20% that are recovered after long periods of rest [1]. Thus, the resilience of the scaffold is a key parameter. Stress-strain curves from compression tests of PEU scaffolds revealed extended elastic regimes of 35% and 25% for 60° and 90° scaffolds, respectively, confirming what we have also reported previously [41]. Cyclic compression of the PEU scaffolds reported here showed an outstanding recovery after 10 cycles, with maximum permanent deformation of 1% for the 60° deposition pattern and only 0.5% for the 90° pattern (Fig. 1). Moreover, it is important to note that quantification of the permanent deformation after each compression cycle revealed the appearance of a plateau after 7 and 8 cycles for the 90° and 60° pattern scaffolds, respectively, indicating that the scaffolds will potentially undergo full shape recovery after further cycles of compression (Fig. S1). Hung et al. [37] reported on the wet additive manufacturing of PEU in combination with 24% poly(ethylene oxide) (PEO) from a water solution for cartilage tissue engineering. Strain recovery tests on fabricated scaffolds showed a permanent deformation of approximately 32% and 43%, for PEU/PEO and PEU (after PEO removal) scaffolds, respectively, after only one compression cycle with 10% strain. Thus, the permanent deformation reported is 43–86 times higher than the one reported here.

PEU materials supported the growth of BMSCs similarly to traditional TCP, with no statistical difference on cell viability and a higher cell attachment after 72 and 24 h. Initial cell attachment on the PEU scaffolds appeared to be dependent on the available surface area, with higher values on scaffolds with higher surface area (90° pattern) (Fig. S3). In long-term culture experiments, however, the cell number remained constant on 90° scaffolds independently of the media used (basal and differentiation, Fig. 2) and slightly increased on scaffolds with a 60° pore architecture, although not statistically significant, which has been already observed by others on additive manufactured scaffolds using BMSCs [26].

In vitro cartilage tissue engineering with BMSCs resulted on a deposition of GAGs that ranged from 2.2 to 2.6 μg GAG/μg DNA (Fig. 2). No significant differences were measured on GAG deposition between the different pore architectures, as previously observed by Di Luca et al. [26]. Surprisingly, the deposition of GAG was neither significantly different between the media conditions used and was, for both scaffold architectures, nor higher than that measured on traditional pellet cultures (Fig. S6). These results seem to indicate that neither the chondrogenic media nor the 3-dimensional environment is responsible for the deposition of GAGs in these particular systems but rather the PEU scaffolds themselves. We recently reported on the in vitro cartilage tissue engineering with these PEU additive manufactured scaffolds and found the same results when a chondrogenic cell line was used, which further supports the idea of these being chondroinductive.

The deposition of collagen, a protein characteristic of cartilaginous tissues, on tissue-engineered scaffolds was also visually examined via histology, revealing no apparent differences after 28 days of culture on scaffolds cultured on basal and differentiation media. However, scaffolds with a 60° pore architecture appeared to be fuller and with a more intense stain, which suggested a higher deposition of collagen (Fig. S7). Immunofluorescence staining of collagen I and collagen II supported this observation. The deposition of collagen II was higher on 60° pore architectures than in 90° ones that only showed intracellular expression of the protein even after 28 days of culture. Moreover, on 60° scaffolds, the deposition of collagen II was observed in both basal and differentiation media conditions. Cell invasion and ECM deposition evaluated from μCT scans further supported this idea, with higher amounts of cell/ECM observed on 60° scaffolds. We hypothesized that the scaffolds with 60° printing pattern presented a most favorable chondrogenic environment due to the lower surface area (as compared with 90° scaffolds) which might promote more cell-cell interactions and reduced focal adhesions on the substrate material, which has been shown to be favorable for chondrogenesis [59]. Indeed, the cell morphology had a rather spindle shape as compared with chondrocytes in their native environment, which is at the same time characteristic of BMSCs cultured on additive manufactured scaffolds [25]. However, similarly to what is observed during cartilage development, we expect that at longer culture times and with a higher ECM deposition, cells would be encapsulated within their own matrix, resulting on the formation of lacunae and acquiring their characteristic rounded morphology.

Differentiation of stem cells toward chondrogenic phenotypes is a dynamic process in which cells undergoing chondrogenic differentiation continue differentiating toward hypertrophic phenotypes, more representative of bony environments and with higher expression of collagen I than that of collagen II. Other markers characteristic of hypertrophic chondrocytes are RunX2 and collagen X, the latter being only present on the deep zone of the articular cartilage. The ratio between the expression of collagen II and collagen I markers is commonly used to characterize the differentiation state of the cells (more chondrogenic when the ratio is higher). Other markers representative of chondrogenesis are Sox5, Sox6, and Sox9, known as the Sox trio, and are necessary for the expression of collagen II [60]. Analysis of the gene expression profile of BMSCs cultured on the PEU scaffolds showed an upregulation of characteristic chondrogenic markers such as collagen II, Sox6, and Sox5, these last two showing similar relative gene expression as Sox9. The upregulation of these chondrogenic markers appeared similar to that of cells cultured on the gold standard pellet culture. Markers of hypertrophy such as collagen X and collagen I were also upregulated, the last two being higher than that in pellet cultures. This is likely to be an indication that the differentiation of the cells is still on a premature state similar to that of in vivo mesenchymal condensation during the development of the osteochondral plate where collagen I is also expressed [61]. Yet, the collagen II/collagen I expression ratio, which is established as a good marker for chondrogenic differentiation [62], of approximately 2–3 was measured after 28 days of culture. Similar values have been reported on the literature for BMSCs cultured on bulk hydrogels that are traditionally considered as a favorable chondroinductive environment, yet too weak to withstand loads applied onto the knee [63].

Analysis of the mechanical properties of the tissue-engineered constructs showed an initial decrease on the Young's modulus of the scaffolds after 14 days of culture, followed by a later increase after 28 days. This trend is common and ascribed to an initial degradation of the polymer followed by an increase due to the deposition of ECM.

A detailed observation of the progression on the gene expression of collagens on cells cultured on both architecture types reveals an unpaired evolution of the chondrogenic markers. While cells cultured on 90° pore architectures showed a progressive increase on the expression of collagen II and decrease on the expression of collagen I and X, cells cultured on 60° pores showed the opposite trend. This is well in line with the observed trend on collagen II and GAG deposition measured for the two types of scaffolds, suggesting that cells cultured on 60° patterns undergo faster chondrogenesis than those cultured on 90° scaffolds. Di Luca et al. [26] who studied the differentiation of BMSCs on different pore architectures reported also higher expression levels of collagen II and aggrecan on scaffolds with a smaller pore angle after 28 days but lower after 14 days, following the same trend presented here and highlighting the importance of pore architecture on cell differentiation. Surprisingly, BMSCs cultured on PEU scaffolds under basal conditions also showed the upregulation of chondrogenic markers.

Analysis of protein deposition and gene expression of BMSCs cultured on PEU scaffolds points to an effective differentiation toward chondrogenic phenotypes. We also observed certain differentiation potential on cells cultured in basal media. This indicates that neither the media nor the 3D culture environment is the unique driver of the chondrogenic potential of the scaffolds but rather the PEU itself. Engler et al. [64] nicely showed the detrimental effect that the matrix elasticity plays on cell differentiation potential and lineage specification. Recently, Olivares-Navarrete et al. [65] showed that this mechanical effect on lineage specification can also be translated to stiffer substrates with mechanical properties that ranged from 0.8 to 309 MPa, which are more characteristic of those of cartilage and bone. Although at this moment it is unclear which is the specific mechanism for the observed chondroinduction here, we hypothesized that the elasticity that this material presents at a macroscopic level is also present at a lower scale. Thus, cells cultured on the surface of PEU are capable of sensing a mechanical environment that is more representative to the one found on native cartilage.

## 5 Conclusions

Altogether, the presented data suggest that the biodegradable PEU used for this study is a good candidate for tissue regeneration of the articular cartilage. The studied scaffolds with 90° and 60° deposition patterns and with a pore size of 500 μm showed a good cell invasion and survival after 14 and 28 days of culture as evidenced by μCT and quantification of DNA (Fig. 2, Fig. 3). The deposition of a specialized ECM rich in GAGs was measured, showing good values for 3D printed materials and was also observed via histological staining (Fig. 2). The deposition of a collagenous matrix was also observed via staining with Picrosirius red (Fig. S7). The specific deposition of collagen II and collagen I was assessed via immunofluorescence (Fig. 3), showing a clear expression of collagen II in all the samples that was larger on 60° patterned scaffolds, suggesting that this structure may also contribute to a higher chondrogenic differentiation of the cells. Analysis of the gene expression profile of cells cultured on PEU scaffolds for 14 and 28 days in differentiation and control conditions showed for both conditions, an upregulation of chondrogenic markers such as collagen II, Sox5, Sox6, and Sox9 (Fig. 4, Fig. 5). Moreover, the gene expression profile of scaffolds in differentiation media after 28 days of culture was similar to that one measured for the gold standard for in vitro chondrogenesis, pellet culture (Fig. 4 and Fig. S9). The mechanical properties of the scaffolds increased over the culture time (Fig. 6), which we ascribed to the deposition of ECM and tissue formation.

## Credit author statement

**Sandra Camarero-Espinosa:** Conceptualization, Methodology, Investigation, Formal analysis, Writing - original draft. **Clarissa Tomasina:** Investigation. **Andrea Calore:** Investigation, Formal analysis. **Lorenzo Moroni:** Conceptualization, Methodology, Writing - review & editing, Supervision, Project administration, Funding acquisition.

## Data availability

All data supporting the findings of this study are available from the corresponding authors upon request.

## Declaration of competing interest

The authors declare that they have no known competing financial interests or personal relationships that could have appeared to influence the work reported in this paper.
