# Supplementary material for: Additive manufactured, highly resilient, elastic, and biodegradable poly(ester)urethane scaffolds with chondroinductive properties for cartilage tissue engineering
Source: Mater Today Bio. 2020 Apr 13;6:100051. doi: 10.1016/j.mtbio.2020.100051 (PMC7229290; doi:10.1016/j.mtbio.2020.100051)
Supplement: Multimedia component 1 [file mmc1.docx]

**Additive Manufactured, Highly Resilient, Elastic and Biodegradable Poly(ester)urethane Scaffolds with Chondroinductive Properties for Cartilage Tissue Engineering.**

Sandra Camarero-Espinosa^1,2^, Clarissa Tomasina^1,2^, Andrea Calore^1,3^ and Lorenzo Moroni^1^.

*^1^MERLN Institute for Technology-inspired Regenerative Medicine, Complex Tissue Regeneration Department, Maastricht University, P.O. Box 616, 6200MD Maastricht, The Netherlands*

*^2^Polyganics, Rozenburglaan 15A,9727 DL Groningen, The Netherlands.*

*^3^Aachen Maastricht Institute for Biobased Materials, Maastricht University, P.O. Box 616, 6200 MD Maastricht, The Netherlands*

**Supporting Information**

Figure S1. Non-cumulative permanent deformation calculated from cyclic compression tests during 10 cycles at 10% strain on scaffolds with 500 µm pore size and a deposition angle of 90° and 60°. Error bars represent standard deviation, n = 5.

Figure S2. Cell number attached to non-coated scaffolds 24h after BMSC seeding. Data represents average ± standard deviation, n = 3. Statistical significance is calculated by the student t-test; (****) p<0.0001, (***) p<0.001, (**) p<0.01 and (*) p<0.1.


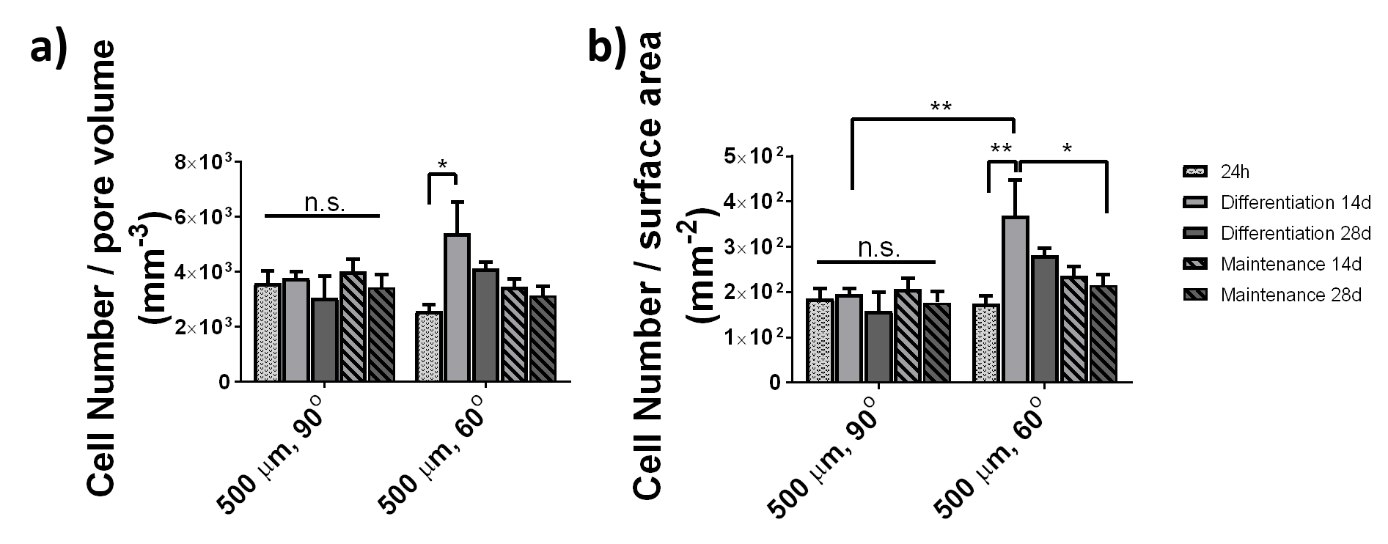


Figure S3. Cell density (a) and cell surface density (b) in scaffolds after 24h of cell seeding and 14 and 28 days in basal and differentiation media. Data represents average ± standard deviation, n = 3. Statistical significance is calculated by the student t-test; (****) p<0.0001, (***) p<0.001, (**) p<0.01 and (*) p<0.1.

Figure S4. Cell number on pellet cultures after 28 days of culture in differentiation and basal (maintenance) conditions. Data is shown as mean ± SD, n = 3. Statistical significance is calculated by the student t-test; (****) p<0.0001, (***) p<0.001, (**) p<0.01 and (*) p<0.1.


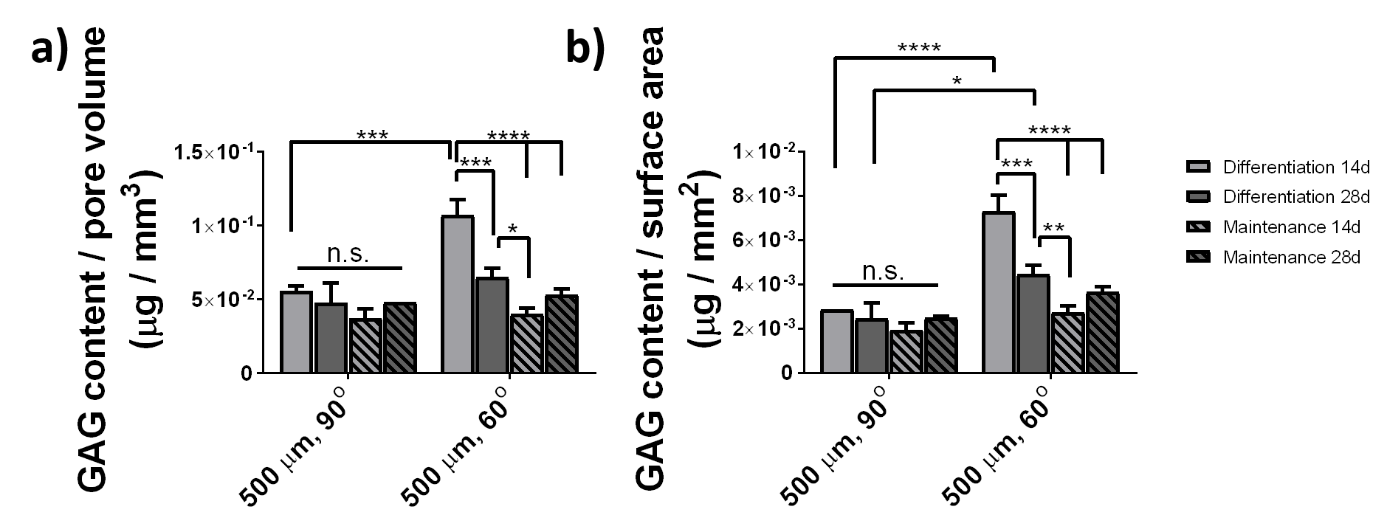


Figure S5. GAG density (a) and GAG surface density on scaffolds after 14 and 28 days of culture on basal and differentiation media. Data represents average ± standard deviation, n = 3. Statistical significance is calculated by the student t-test; (****) p<0.0001, (***) p<0.001, (**) p<0.01 and (*) p<0.1.

Figure S6. Normalized GAG content on pellet cultures after 28 days of culture in differentiation and basal (maintenance) conditions. Data is shown as mean ± SD, n = 3. Statistical significance is calculated by the student t-test ; (****) p<0.0001, (***) p<0.001, (**) p<0.01 and (*) p<0.1.


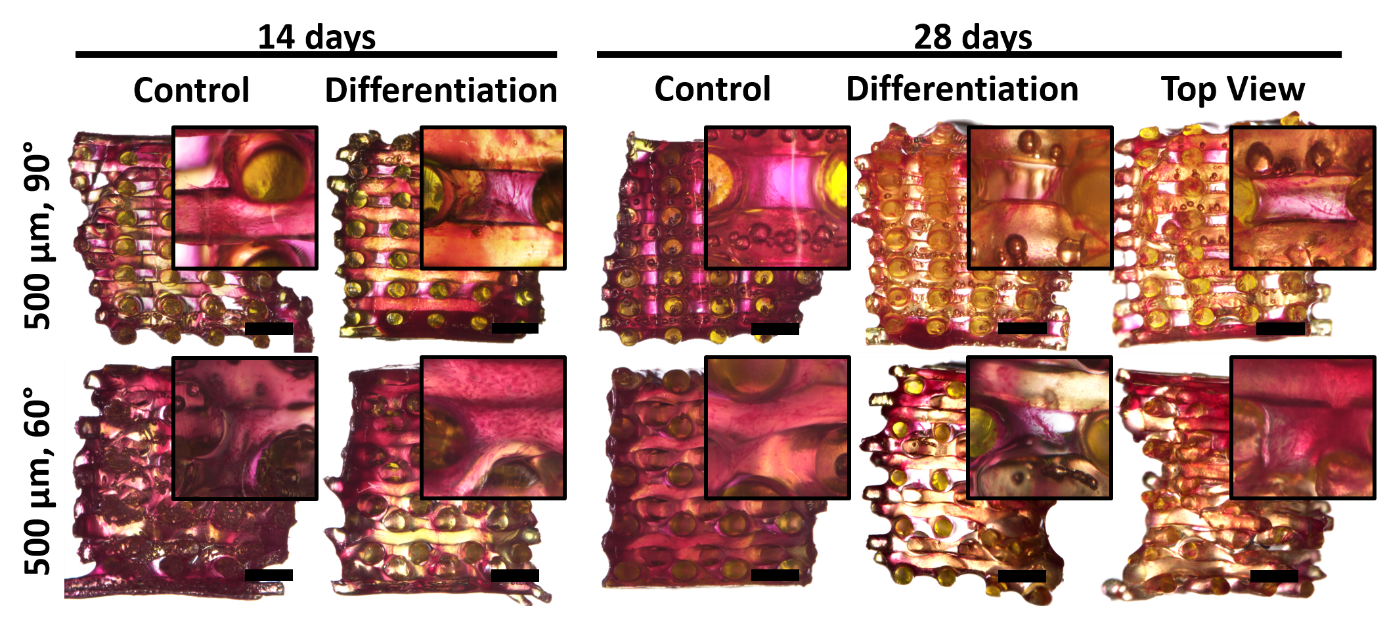


Figure S7. Collagen deposition. Optical microscopy images of scaffolds cultured for 14 and 28 days in differentiation and basal (control) media and stained with Picrosirius red. Top view column shows the outer perimeter of scaffolds cultured for 28 days in differentiation media. Scale bar is 1mm, insets are 735x735 µm.


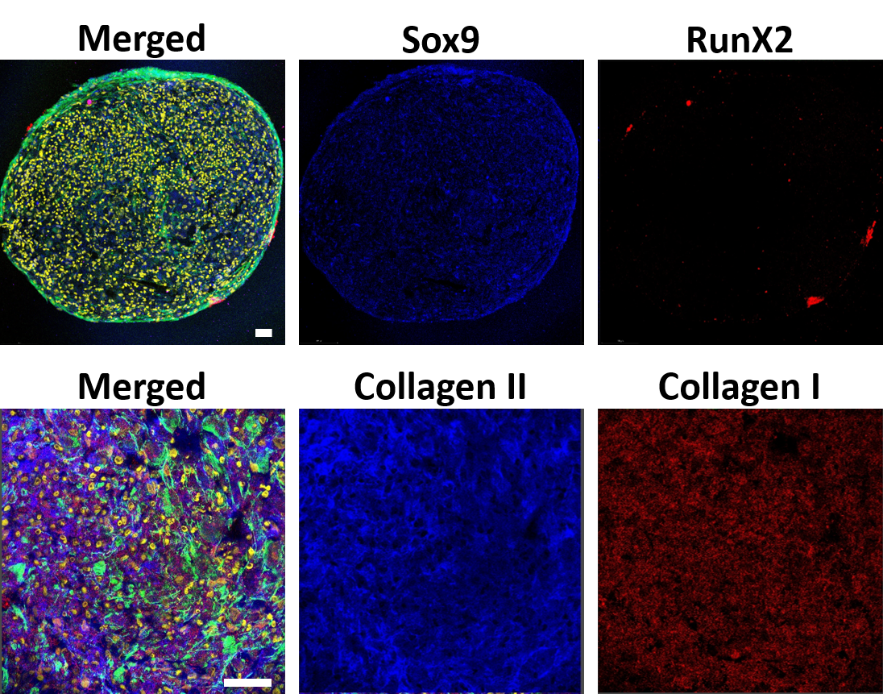


Figure S8. Light scanning microscopy (LSM) images of cell pellet after 28 days of culture in differentiation media. Cells were stained for F-Actin (Phalloidin, green), DNA (Hoechst, yellow), collagen II (blue) and collagen I (red) (d). Scale bars are 50 µm.


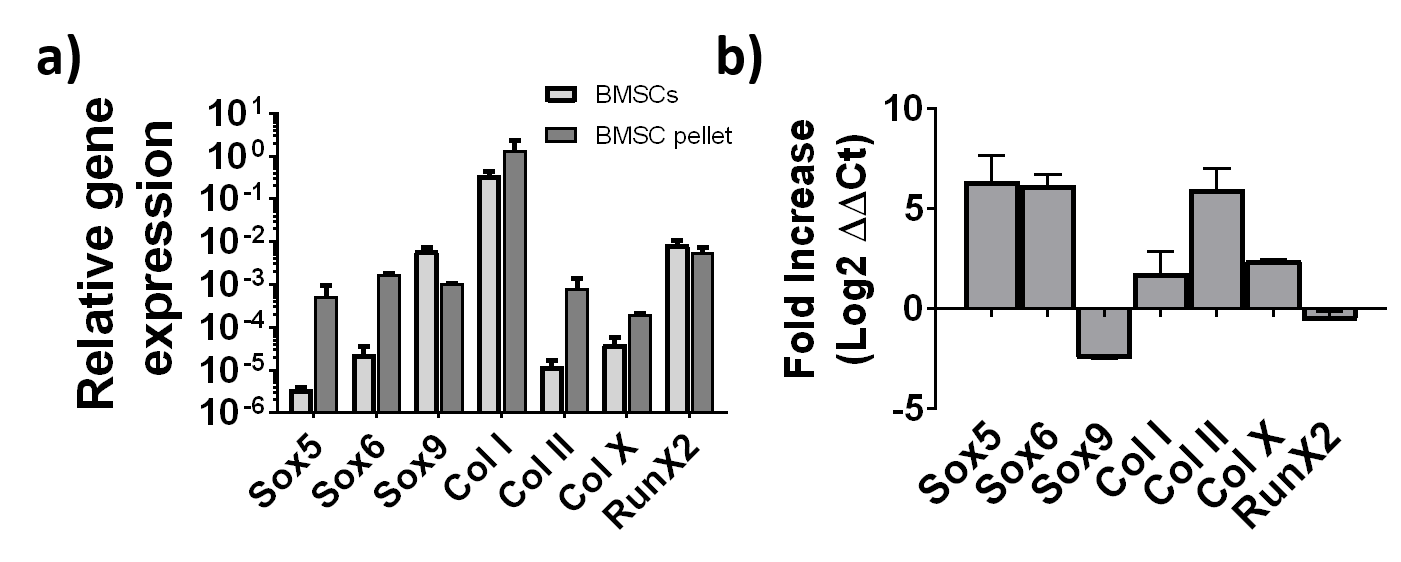


Figure S9. Relative gene expression of BMSCs on monolayer culture and differentiated cells after 28 days of pellet culture (a) and the calculated fold increase on gene expression (b). Error bars represent standard deviation.
